# Antibody response of a particle-inducing, liposome vaccine adjuvant admixed with a Pfs230 fragment

**DOI:** 10.1038/s41541-020-0173-x

**Published:** 2020-03-18

**Authors:** Wei-Chiao Huang, Bingbing Deng, Amal Seffouh, Joaquin Ortega, Carole A. Long, Ragavan V. Suresh, Xuedan He, Kazutoyo Miura, Shwu-Maan Lee, Yimin Wu, Jonathan F. Lovell

**Affiliations:** 1 Department of Biomedical Engineering, University at Buffalo, State University of New York, Buffalo, NY 14260 USA; 2 Laboratory of Malaria and Vector Research, National Institute of Allergy and Infectious Diseases, National Institutes of Health, Rockville, MD 20852 USA; 3 Department of Anatomy and Cell Biology, McGill University Montreal, QC, H3A 0C7 Canada; 4 PATH’s Malaria Vaccine Initiative (MVI), Washington, DC 20001 USA

**Keywords:** Biotechnology, Immunology

## Abstract

Pfs230 is a malaria transmission-blocking antigen candidate, expressed on the surface of *Plasmodium falciparum* gametocytes. A recombinant, his-tagged Pfs230 fragment (Pfs230C1; amino acids 443–731) formed serum-stable particles upon incubation with liposomes containing cobalt-porphyrin-phospholipid (CoPoP). In mice, immunization with Pfs230C1, admixed with the adjuvants Alum, Montanide ISA720 or CoPoP liposomes (also containing synthetic monophosphoryl lipid A; PHAD), resulted in elicitation of IgG antibodies, but only those induced with CoPoP/PHAD or ISA720 strongly reduced parasite transmission. Immunization with micrograms of Pfs230C1 adjuvanted with identical liposomes lacking cobalt (that did not induce particle formation) or Alum was less effective than immunization with nanograms of Pfs230C1 with CoPoP/PHAD. CoPoP/PHAD and ISA720 adjuvants induced antibodies with similar Pfs230C1 avidity but higher IgG2-to-IgG1 ratios than Alum, which likely contributed to enhanced functional activity. Unlike prior work with another transmission-blocking antigen (Pfs25), Pfs230C1 was found to be effectively taken up by antigen-presenting cells without particle formation. The anti-Pfs230C1 IgG response was durable in mice for 250 days following immunization with CoPoP/PHAD, as were antibody avidity and elevated IgG2-to-IgG1 ratios. Immunization of rabbits with 20 µg Pfs230C1 admixed with CoPoP/PHAD elicited antibodies that inhibited parasite transmission. Taken together, these results show that liposomes containing CoPoP and PHAD are an effective vaccine adjuvant platform for recombinant malaria transmission blocking antigens.

## Introduction

Malaria is caused by *Plasmodium* parasites and is transmitted to and from humans by female *Anopheles* mosquitoes. Transmission-blocking vaccines (TBVs), which block the transmission of parasites to human hosts, have been proposed as a strategy to mitigate disease spread^1–3^. TBVs aim to prevent transmission of parasites to other humans following a mosquito blood meal from an infected but TBV-immunized human. The host should generate circulating antibodies that, upon entering the mosquito midgut, block gametocyte fertilization^4^ and/or prevent oocyte development^5^. An effective TBV should induce long-lasting, elevated levels of functional antibodies in the host, and antibody transfer to mosquitos during a blood meal is generally considered the most viable functional mechanism.

Pfs230 is a transmission-blocking antigen expressed on the surface of gametocytes within human red blood cells, so the host immune responses have potential to be re-boosted by natural infection, unlike some other TBV antigens like Pfs25, which is expressed only in mosquito hosts^6^. On the other hand, presence in the host could drive development of parasite immune escape mechanisms. However, gametocytes are located in red blood cells, so can hide within host cells^7^. Since red blood cells do not express major histocompatibility complexes on their surface and are not recognized by T cells, the parasite is likely to avoid clearance by the host immune response^8^. Pfs230 contains 3135 amino acids, and due to this large size, the full-length protein is challenging to produce in recombinant systems^4^. An exhaustive study of Pfs230 fragments spanning the entire protein suggested that only constructs containing the first cysteine motif domain (amino acids 589–730) induce transmission-reducing activity^9^. Insect cell-based production of a recombinant his-tagged N-terminal fragment of Pfs230 (amino acids 443–731), termed Pfs230C1, which induces transmission-reducing antibodies in mice, has been described previously, and was the antigen used in this study^10^.

IgG subclass influences transmission-blocking activity of anti-Pfs230 antibodies^11^. Anti-Pfs230 mouse monoclonal antibodies with transmission blocking activity have been identified for the IgG2 subclass but not the IgG1 subclass^12^. Thus, a higher IgG2-to-IgG1 ratio may be important for maximal transmission-blocking activity of anti-Pfs230 antibodies. A Pfs230 fragment with exoprotein A carrier protein (EPA) formulated with the liposomal adjuvant AS01 has been used in Phase I clinical trials (NCT02942277). AS01 contains monophosphoryl lipid A (MPLA) and QS-21 in a liposome vehicle. MPLA is a toll-like receptor-4 (TLR-4) agonist that is non-pyrogenic form of lipopolysaccharide^13^. MPLA can activate antigen-presenting cells for enhanced immune responses^14^. A synthetic version of MPLA, PHAD, which is a pure compound (as opposed to a mixture of lipids), was incorporated into the liposomes used in this study.

Vaccine adjuvants are often combined with recombinant antigens to enhance immune responses. Alum is the most common adjuvant for recombinant antigens and has a successful track record in human vaccination^15^. Other more potent adjuvants have been developed including Montanide ISA51 and ISA720; water in oil emulsions that have been tested in humans^16^. A prior trial with the TBV antigen Pvs25 with ISA51 found frequent local reactogenicity and some systemic adverse events including two cases of erythema nodosum^17^. ISA720 is considered more potent than ISA51, and therefore it is not clear whether ISA720 would be suitable for wide-scale human use in a prophylactic vaccine. Liposomes containing cobalt-porphyrin-phospholipid (CoPoP) and PHAD have not yet been tested in humans, but have been proposed as a next-generation vaccine adjuvant, and were shown to be effective with his-tagged Pfs25, in mice and rabbits^18^. That study showed that Pfs25 could be admixed with CoPoP/PHAD liposomes for so-called spontaneous nanoliposome-antigen particleization (SNAP), a unique mechanism of action for a vaccine adjuvant, which induced strong transmission-reducing activity following immunization. Recombinant his-tagged antigens without further modification can readily bind CoPoP/PHAD liposomes for enhanced immune responses. Other related approaches for conversion of soluble TBV antigens to particle-based vaccines also show promise^19–23^. Pfs230 fragments (amino acids 542–736) have also been shown to be effective when bioconjugated to protein toxins and outer membrane protein complexes^24,25^. In this study, immunization with Pfs230C1 in mice and rabbits is assessed with CoPoP/PHAD as a particle-inducing vaccine adjuvant.

## Results

### Pfs230C1 spontaneously binds to CoPoP liposomes

**Fig. 1:**
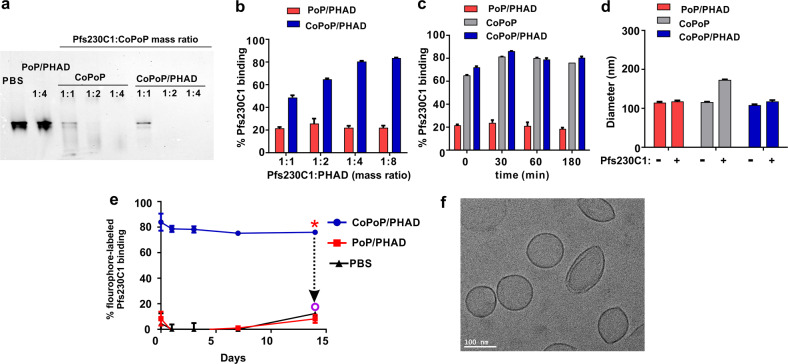
His-tagged Pfs230C1 spontaneously forms particles when admixed with liposomes containing CoPoP. **a** Native PAGE of Pfs230C1 after 1 h incubation with indicated liposomes. The antigen:liposome mass ratio (on CoPoP or PoP basis) is indicated and 1.5 µg of Pfs230C1 was loaded in each lane. The absence of a protein band is indicative of antigen binding to the liposomes, which are too large to migrate in the gel. Representative of three independent experiments. **b** Binding of Pfs230C1 with varying amounts of CoPoP/PHAD or PoP/PHAD liposomes based on microcentrifugal filtration. **c** Pfs230C1 binding kinetics based on microcentrifugal filtration. **d** Size of liposomes before and after Pfs230C1 binding, based on dynamic light scattering. **e** Serum stability of CoPoP/PHAD with fluorophore-labeled Pfs230C1 during incubation for 2 weeks in 20% human serum at 37 ˚C. The asterisk shows the time after which 0.1% Triton X-100 and proteinase K were added. Graphs show mean ± std. dev. for *n* = 3 independent experiments. **f** Cryo EM image of Pfs230C1 with CoPoP/PHAD. Image was taken from a single experiment.

CoPoP liposomes particleize his-tagged antigens with simple mixing^26^. To assess the binding of his-tagged Pfs230C1 to CoPoP liposomes, native polyacrylamide gel electrophoresis (PAGE) was used (Fig. 1a; the full blot is shown in Supplementary Fig. 1). 1.5 µg of Pfs230C1 was loaded into each lane of the gel and then subjected to electrophoresis. The native gel system does not contain detergents, therefore, if the antigen is bound to liposomes, it becomes too large to enter the gel. The absence or presence of protein migration into the gel following particle formation indicates that Pfs230C1 bound to liposomes containing CoPoP, but not to otherwise identical liposomes containing cobalt-free porphyrin-phospholipid (PoP). Increasing mass ratios of CoPoP:Pfs230C1 from 1:1 to 4:1 resulted in increased binding of the antigen. This was consistent with an independent microcentrifugal filtration binding assay which assesses the fraction of non-bound protein that can pass through a membrane filter, as the liposomes are too large to do so. A 4:1 mass ratio of CoPoP to Pfs230C1 achieved 80% binding of the antigen (Fig. 1b). Minimal Pfs230C1 binding was observed when mixed with PoP/PHAD liposomes lacking cobalt. Binding kinetics showed rapid binding, with approximately 80% protein bound to CoPoP liposomes at room temperature within 1 h (Fig. 1c). Based on dynamic light scattering, the liposomes with particleized Pfs230C1 were approximately 100 nm in diameter, and minimal change in size was induced by antigen binding (Fig. 1d). Owing to the visible light absorption of CoPoP, fluorescence resonance energy transfer (FRET) assays are able to report on the binding of fluorescently labeled antigens. Throughout two weeks of incubation in 20% bovine serum at 37 °C, fluorophore-labeled Pf230C1 binding to CoPoP/PHAD liposomes remained intact, whereas no binding was observed with PoP/PHAD liposomes (Fig. 1e). The fluorescence of the dye label could be restored upon dissociating the liposomes with 0.1% Triton-X100 detergent and digesting the protein with proteinase K. Cryo-electron microscopy (cryo-EM) with Pfs230C1 bound to CoPoP/PHAD liposomes showed particles that exhibited an either spherical or slightly oblong shape. Their length for most of the observed particles was approximately 100 nm (Fig. 1f). Pfs230C1 itself was not large enough to be visualized on the liposome surface. Taken together, these results shows that the Pfs230C1 antigen rapidly forms 100 nm particles after admixing with CoPoP liposomes.

### Pfs230C1 immunization with CoPoP/PHAD induces functional IgG

**Fig. 2:**
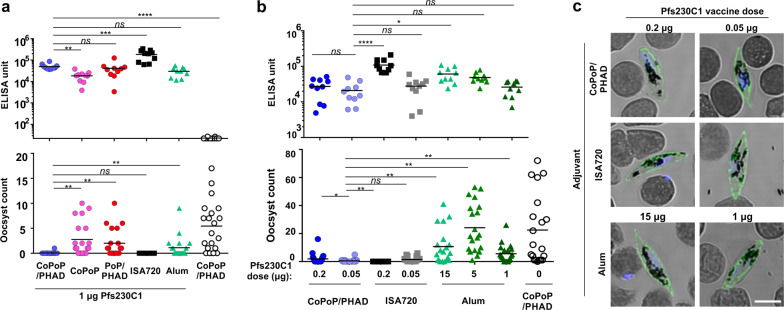
Pfs230C1 adjuvanted with CoPoP/PHAD or ISA720, but not Alum, induces IgG with strong transmission-reducing activity. **a**, **b** Anti-Pfs230C1 IgG ELISA data (top) and standard membrane feeding assay functional assay (SMFA, bottom). **a** ICR mice were immunized with 1 μg of Pfs230C1 with indicated adjuvants. **b** ICR mice were immunized with micrograms of Pfs230C1 with Alum or nanograms of Pfs230C1 with CoPoP/PHAD or ISA720. ELISA experiments in (**a**, **b**) were performed with *n* = 10 independent mice; lines show geometric mean, while SMFA experiments were performed with *n* = 20 mosquitoes; lines show arithmetic mean. Black open circles in (**a**, **b**) represent control mice immunized with CoPoP/PHAD alone, without Pfs230C1. **c** Immunofluorescence assay of NF54 parasites at gametocyte stage. Images taken from a single experiment. Scale bar, 10 μm. For (**a**, **b**), log10-transformed titer were analyzed by one-way ANOVA test followed by Tukey’s comparisons. *p < 0.05; **p < 0.01; ***p < 0.001; ****p < 0.0001. For SMFA functional assay, the statistical differences were analyzed by a zero-inflated negative binomial model^38^.

To investigate whether immunization with the particleized antigen could generate antibodies against Pfs230C1, outbred mice were vaccinated intramuscularly with 1 μg of Pfs230C1, admixed prior to injection with various adjuvants. These included the liposomal adjuvants of CoPoP/PHAD, CoPoP, or PoP/PHAD, then also Alhydrogel (Alum) or Montanide ISA720. As shown in Fig. 2a and Supplementary Table 1 (study number MA0068-1), all the immunization groups resulted in anti-Pfs230C1 IgG production. CoPoP/PHAD induced higher levels of antibodies compared to CoPoP alone, lower levels of antibodies compared to ISA 720, and similar levels of antibodies compared to Alum and non-particleizing PoP/PHAD liposomes. Despite the similar overall antibody levels, the groups vaccinated with CoPoP/PHAD liposomes and ISA720, but not Alum or PoP/PHAD liposomes induced strong transmission-blocking activity in a standard membrane feeding assay (SMFA). As shown in the first 3 rows of Supplementary Table 1 (study number MA0068-1), and as discussed further below, CoPoP/PHAD liposomes induced antibodies with substantially higher IgG2-to-IgG1 ratios compared to Alum, but with similar avidity. For this analysis, different antigen doses are included, but only immunization conditions which resulted in anti-Pfs230C1 ELISA units (EU) greater than 2500 were considered (*n* = 9 for Alum and *n* = 10 for CoPoP/PHAD). Inclusion of PHAD along with CoPoP also appeared to increase the IgG2-to-IgG1 ratio (1st vs. 4th row of Supplementary Table 1, MA0068-1). CoPoP/PHAD liposomes induced antibodies with only marginally higher IgG2-to-IgG1 ratios and moderately higher avidity compared PoP/PHAD liposomes that lacked cobalt. However, compared to PoP/PHAD which did not effectively bind Pfs230C1, CoPoP/PHAD liposomes induced antibodies with significantly better transmission-reducing activity. The mechanism for this phenomenon is not clear, but co-delivery of the antigen along with the PHAD adjuvant to the same location and immune cells may result in stimulation that gives rise to better antibody development.

Additional studies were then carried out to gain further investigate these observations. First, mice were immunized with adjusted doses of Pfs230C1 with CoPoP/PHAD, ISA720 and Alum adjuvants. Since 1 μg Pfs230C1 did not elicit strongly functional antibodies with Alum, the dose of Pfs230C1 was increased to 15 μg. Conversely, since CoPoP/PHAD liposomes and ISA720 generated strong functional antibodies, the Pfs230C1 dose was lowered from 1 μg to 200 and 50 ng. As shown in Fig. 2b, mice immunized with nanograms of Pfs230C1 with CoPoP/PHAD or ISA720 induced slightly lower IgG titers albeit with no statistical difference compared to mice immunized with micrograms of Pfs230C1 with Alum. Again, antibodies induced by mice immunized with Alum did not have strong transmission-blocking activity at 15 μg of Pfs230C1 compared to CoPoP/PHAD or ISA720 with nanogram dosing (Fig. 2b, Supplementary Table 1; MA-0086). The average number of oocysts were significantly lesser comparing CoPoP/PHAD to Alum, as well comparing ISA720 to Alum. In order to determine whether these antibodies could bind Pfs230 on the surface of gametocytes, an indirect immunofluorescence assay (IFA) was performed. It has previously been shown that the Pfs230C1 antigen, when immunized at 16.5 μg with ISA720, induces reactivity with gametocytes with IFA^10^. As shown in Fig. 2c, the antibodies were reactive with gametocytes in the IFA, as observed with microscopy. Thus, the IgG induced by all three adjuvants recognized native Pfs230 on the surface of parasites. In summary, microgram dosing Pfs230C1 with Alum induced high levels of antibodies that recognized the parasite, but that had marginal efficacy in the SMFA. Nanogram dosing of Pfs230C1 with CoPoP/PHAD and ISA720 resulted in antibodies with strong function in the SMFA.

### Particleization is not required for Pfs230C1 uptake by antigen-presenting cells

**Fig. 3:**
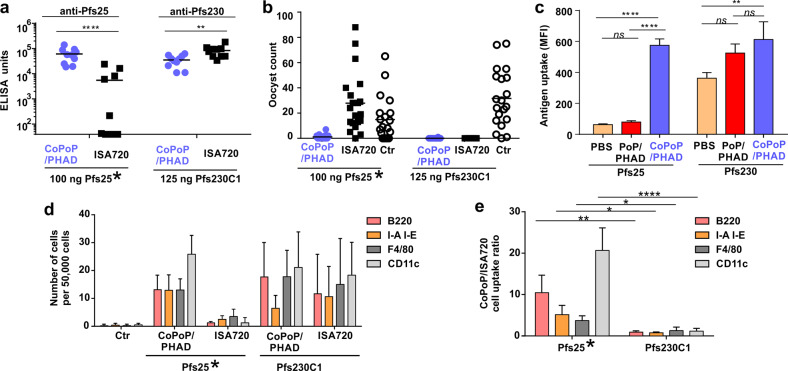
Pfs230C1, but not Pfs25, is taken up by APCs without particleization and is effectively adjuvanted by Montanide ISA720. Anti-Pfs230C1 and anti-Pfs25 IgG ELISA (**a**) and SMFA (**b**) with indicated antigen adjuvanted with CoPoP/PHAD or ISA720. **c** RAW264.7 macrophage uptake of fluorophore-labeled Pfs25 or Pfs230C1 with indicated adjuvant assessed as mean fluorescence intensity (MFI) using flow cytometry. **d** Lymph node uptake of antigens into APCs following intramuscular injection. **e** Uptake ratio of antigens adjuvanted with CoPoP over ISA720 in lymph nodes. ELISA experiment in **a** were performed with *n* = 10 independent mice and lines show geometric mean. SMFA experiments in (**b**) were performed with *n* = 20 mosquitoes and lines show arithmetic mean. Bar graphs show mean±std. dev. for *n* = 3 independent experiments. Pfs25 asterisks in a, b, d and e indicate data re-use that was previously reported in ref. ^18^. For (**a**), the titer comparison between CoPoP/PHAD and ISA720 with indicated proteins, log-transformed data was compared with Student's *t* test. For (**c**), one-way ANOVA followed by Tukey’s comparison was used. For **e**, the comparison between CoPoP/ISA720 ratio uptake in APC was compared with Student's *t* test. *p < 0.05, **p < 0.01; ****p < 0.0001.

Our previous study on Pfs25 showed that compared to other vaccine adjuvants (e.g., ISA720, AS01-like liposomes, PoP/PHAD liposomes, Alum, etc.) only the CoPoP/PHAD approach induced uptake by antigen-presenting cells (APCs) and produced a strong immune response^18^. This behavior could be explained due to the propensity for APCs to efficiently take up particles, like 100 nm liposomes, but not free Pfs25. In contrast to prior immunogenicity findings with Pfs25, Pfs230C1 induced antibodies when combined with various different adjuvants (Fig. 2). To probe the differences in immunogenic behavior between Pfs25 and Pfs230C1, a comparison of IgG titer and SMFA activity between Pfs25 to Pfs230C1 was assessed, focusing on CoPoP/PHAD and ISA720 adjuvants (Fig. 3a, b and Supplementary Table 2) Mice immunized with CoPoP/PHAD and Pfs25 or Pfs230C1 induced strong SMFA activity. On the other hand, ISA720 only induced strong SMFA activity with Pfs230C1, but not with Pfs25 at the nanogram immunization dose used. To account for these differences, macrophage uptake of both Pfs25 and Pfs230C1 was compared in vitro, the cells were incubated with fluorophore-labeled Pfs25 or Pfs230C1 with CoPoP/PHAD liposomes or PoP/PHAD liposomes. Fig. 3c and Supplementary Fig. 2 show that Pfs25 was effectively taken up by macrophages when converted into particulate form with CoPoP/PHAD, but not as a soluble antigen. In contrast, Pfs230C1 was uptaken by macrophages even without being bound to liposomes. We next examined whether uptake of Pfs230C1 in APCs also occurred in vivo in draining lymph nodes. As shown in Fig. 3d, 2 days following immunization with CoPoP/PHAD or ISA720, Pfs230C1 was detected in major types of APCs, including B-cells (B220), macrophages (F4/80), dendritic cells (CD11c) and MHCII-expressing cells (I-A/I-E) with either the CoPoP/PHAD liposomes or ISA720. The gating of APC cells is shown in Supplementary Fig. 3a–d. This, again, is different compared to Pfs25, which was only taken up by APCs in draining lymph nodes when admixed with CoPoP/PHAD, and uptake was limited when combined with ISA720. Fig. 3e shows that for Pfs25, CoPoP/PHAD was more effective than ISA720 at delivering the antigen to immune cells in vivo (as determined by the differential antigen uptake ratio between the two adjuvants), suggesting that sufficient antigen delivery to APCs is an important factor for inducing transmission blocking antibodies. Uptake of Pfs25 into varying immune cell types was over an order of magnitude higher when CoPoP liposomes were used, relative to ISA720. However, for Pfs230C1, both adjuvants worked effectively, resulting in an inter-adjuvant antigen uptake ratio closer to unity. Taken together, these data show that enhanced antigen uptake can explain, at least in part, why only CoPoP liposomes were an effective vaccine adjuvant for Pfs25, but why both CoPoP liposomes and ISA720 were effective for Pfs230C1.

### High IgG2-to-IgG1 ratios in mice immunized with Pfs230C1 and CoPoP/PHAD liposomes

**Fig. 4:**
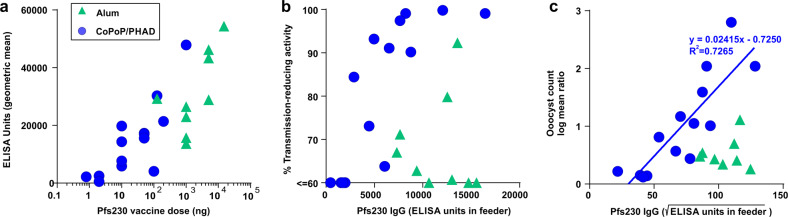
Dose dependent induction of IgG and transmission-reducing activity following immunization of Pfs230C1 with CoPoP/PHAD liposomes. **a** IgG titer as a function of Pfs230C1 injection doses in mice. All dots represented geometric mean titers from *n* = 10 mice. **b** Percentage of TRA to anti-Pfs230 IgG level in feeder and (**c**) LMR to anti-Pfs230C1 IgG level in feeder (square root scale). Each dot represents purified IgG from *n* = 10 mice. Oocytes were counted and percentage of transmission-reducing activity or the log mean ratio of the oocyst count (of the test sample compared to the positive control) were calculated from *n* = 20 mosquitos.

We next examined the differential immune response between Alum and CoPoP/PHAD, since both adjuvant with Pfs230C1 induced high levels of IgG, yet only Pfs230C1 with CoPoP/PHAD had strong SMFA functional activity. Several independent immunization studies at varying dose levels were carried out (Supplementary Table 1). 14 groups of mice (*n* = 10 for each group) were immunized with Pfs230C1 and CoPoP/PHAD and nine groups of mice (*n* = 10 for each group) immunized with Pfs230C1 and Alum. As shown in Fig. 4a, increasing immunization doses of Pfs230C1 resulted in increasing serum IgG EU for Pfs230C1 antibodies for both CoPoP/PHAD and Alum adjuvants. To assess antibody function, SMFA activity of purified serum IgG, in terms of the transmission-reducing activity, is plotted against the amount of specific antibody present (in terms of EU) in Fig. 4b. All data points correspond to separate immunization and SMFA experiments. At higher EU, greater SMFA was only apparent with the CoPoP/PHAD post-immune antibodies. Alum samples had relatively low transmission-reducing activity regardless of the amount of antibody present. The same data is shown in Fig. 4c as the log mean oocyst ratio (LMR) as a function of the square root of EU in the feeder, which has been shown to follow a linear relationship^11^. Although the CoPoP/PHAD data could be reasonably fit with a linear equation (indicated on the graph), Alum did not exhibit such a correlation, with consistently weak transmission-reducing activity. These results imply that CoPoP/PHAD with Pfs230C1 induced better quality of antibodies that effectively block transmission, compared to Alum, which induced less functional Pfs230C1 antibodies.

**Fig. 5:**
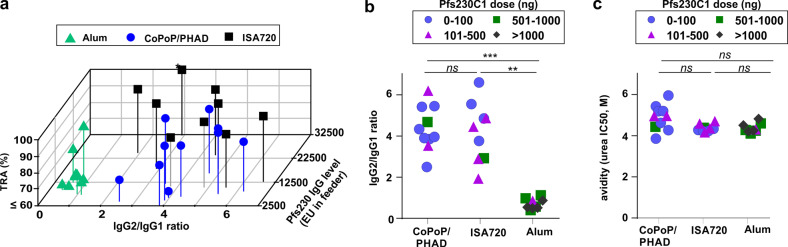
Effects of Anti-Pfs230C1 IgG subclass and avidity induced with Alum, CoPoP/PHAD and ISA720 adjuvants. **a** Three-dimensional plot showing impact of IgG2-to-IgG1 ratios and anti-Pfs230 IgG ELISA units (EU) in the feeder on transmission-reducing activity (TRA). Each point represents an independent experiment using the purified serum IgGs from 10 immunized mice and assessing TRA in 20 mosquitos. The asterisk shows a point that has an EU greater than 32500 (the actual EU was 39613). All percent TRA values less than 60% are shown as 60% in (**a**). Only values with EU greater than 2500 are shown. The (**b**) IgG2-to-IgG1 ratio and (**c**) avidity are shown. Each point reperesents the purified IgG from *n* = 10 mice. For (**b**) and (**c**), the Kruskal–Wallis test followed by Dunn’s multiple comparisons test were used for statistical analysis. **p < 0.01; ***p < 0.0005.

Further studies were carried out to investigate whether the functional activity of antibodies was related to IgG subclass and avidity, As shown in Fig. 5a, higher IgG2-to-IgG1 ratio could at least in part explain the stronger SMFA activity of CoPoP/PHAD relative to Alum. Alum Pfs230C1 antibodies had a low IgG2-to-IgG1 ratio and had weak SMFA activity. Fig. 5b shows that CoPoP/PHAD liposomes and ISA720 induced mainly IgG2 subclass antibodies in mice immunized with varying amount of Pfs230C1, the injection dose of Pfs230C1 did not correlate with the IgG2-to-IgG1 ratio. As shown in Fig. 5c, the avidity of the Pfs230C1 antibodies was similar among all the adjuvant groups. PHAD has been tested in several mouse models and reported to induce stronger IgG2 titers^18,27,28^, which has similar immune response as ISA720^29^. On the other hand, Alum has been reported to generate higher levels IgG1 antibodies^30^. This could be one explanation why Alum could induce Pfs230C1 specific antibodies with weak SMFA activity.

### Durability of CoPoP/PHAD immunization

**Fig. 6:**
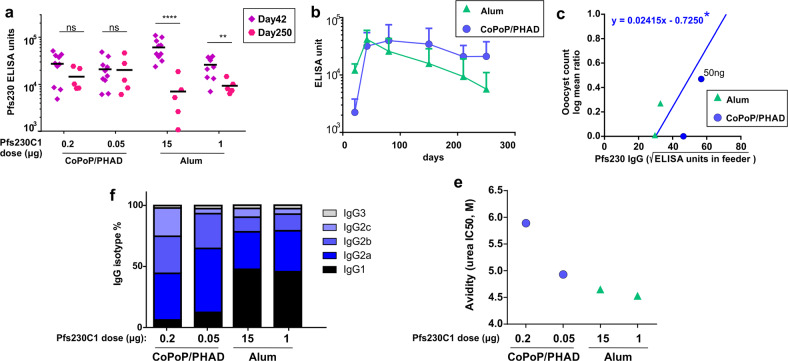
Durability of the anti-Pfs230C1 IgG response. Mice were immunized intramuscularly with 100 ng Pfs230C1 admixed with CoPoP/PHAD liposomes (400 ng PHAD) and 15 and 1 μg of Pfs230C1 with Alum on day 0 and 21. **a** Comparison of IgG titer between day 42 (*n* = 10 mice) and day 250 (*n* = 5 mice). **(b)** Antibody kinetics for Alum (15 ug Pfs230C1) and CoPoP/PHAD (50 ng Pfs230C1). Geometric mean and 95% confidence interval of groups (n = 5 mice) are shown. **(c)** LMR to EU in feeder (square root scale) with day 250 IgGs. Each dot represents purified IgG from *n* = 10 mice. The asterisk shows the same best-fit line using day 42 CoPoP/PHAD data shown in Fig. 4c. **(d**) IgG subclass percentage of indicated adjuvants with different amount of Pfs230C1. **(e)** avidity of indicated adjuvants with different amount of Pfs230C1, each dot represents purified IgG from *n* = 10 mice. For **a**, the titer comparison between Day 42 and Day250 with indicated adjuvants, log10-transformed titer were analyzed by Student's *t* test.

To assess long-lasting immune responses, mice were immunized with Pfs230C1 admixed with CoPoP/PHAD or Alum and then monitored for 250 days. As shown in Fig. 6a, following CoPoP/PHAD immunization with Pfs230C1 with injections on day 0 and day 21, the antibody levels at the end of the study (day 250) did not decrease to less than half of the day 42 levels. There was not a statistically significant decrease in the antibody levels from day 42 to day 250 for mice immunized with the CoPoP/PHAD adjuvant and a 50 ng Pfs230C1 dose (*p* = 0.78, based on student’s *t* test of log-transformed ELISA units) nor a 200 ng Pfs230C1 dose (*p* = 0.25). On the other hand for the groups immunized with Alum, the anti-Pfs230C1 titer significantly decreased by 64%, for the 1 μg Pfs230C1 antigen dose (*p* = 0.0051) and by 88% for the 15 μg antigen dose (*p* < 0.0001). As shown in Fig. 6b, the antibody kinetics in mice over that period showed that the Alum with 15 μg of Pfs230C1 had a steady decrease in antibody levels, whereas mice receiving CoPoP/PHAD with 50 ng Pfs230C1 maintained sustained high levels throughout.

On day 250, we observed modest transmission-reducing activity (TRA) in some groups, with 66% TRA for the 50 ng Pfs230C1 with CoPoP group and 47% TRA for the 1 µg Pfs230C1 with Alum group. All IgG samples were tested at 750 μg/mL IgG, the same total IgG concentration as the day 42 samples (Supplementary Table 1, study number MA0068-1). For the CoPoP/PHAD groups, unlike the geometric mean of the IgGs in serum, the anti-Pfs230C1 levels per total IgG following IgG pooling and purification were lower on day 250 than those in day 42 IgGs (4.1- and 2.4-time lower in 200 and 50 ng of Pfs230C1 group, respectively). The reason for this was not clear. Therefore, to compare the quality of anti-Pfs230C1 antibodies (i.e., whether the same amount of anti-Pfs230C1 antibody shows the same level of activity in SMFA), we plotted the oocyst log mean ratio against anti-Pfs230 IgG level in the feeder (square root) for day 250 data, and compared with the best-fit linear line calculated with multiple IgGs from CoPoP/PHAD adjuvant groups shown in Fig. 4c. As presented in Fig. 6c, the day 250 data did not differ substantially from the best-fit line. Furthermore, after 250 days, CoPoP/PHAD induced strong IgG2 subclass of antibodies and the avidity of IgGs was unchanged (Fig 6d, e and Supplementary Table 1, study number MA-0086-00) Taken together, the decrease of SMFA activity in CoPoP/PHAD with Pfs230C1 on day 250 was likely due to the decrease in proportion of anti-Pfs230C1-specific antibody among total IgGs following pooling and purification. The long-lived antibody response observed in mice immunized with Pfs230C1 admixed CoPoP/PHAD liposomes is consistent with our observations for other antigens including Pfs25^18^ and OspA^31^.

**Fig. 7:**
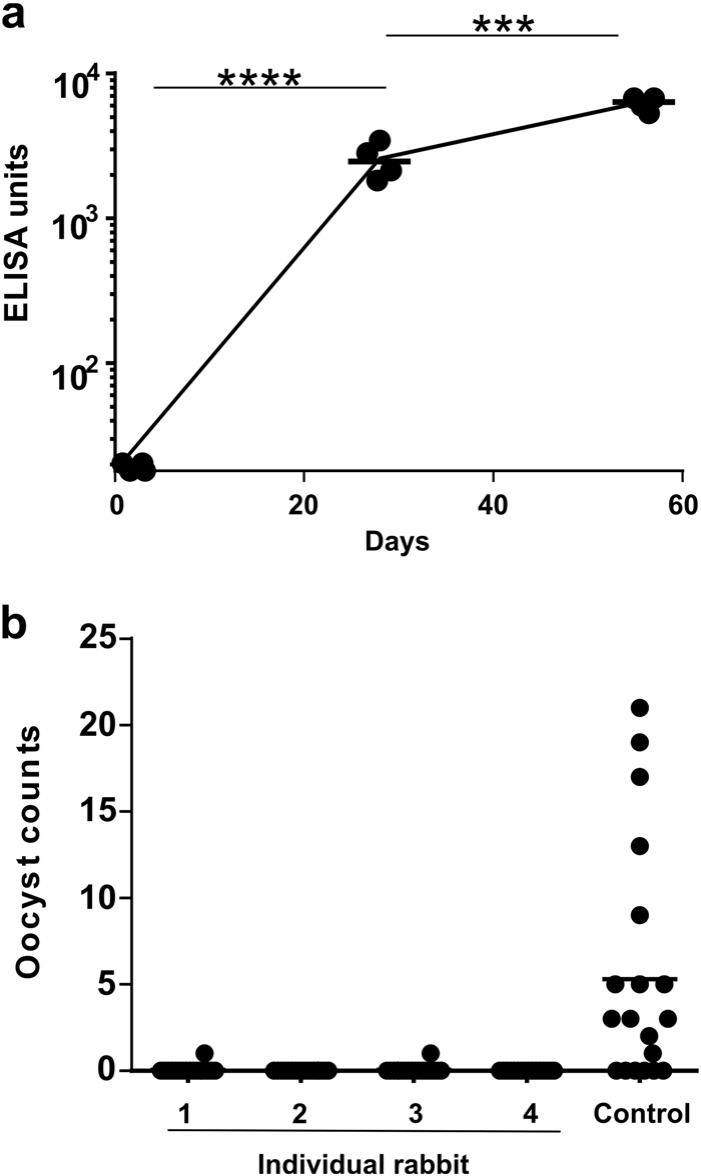
Transmission blocking activity in rabbits induced by Pfs230C1 admixed with CoPoP/PHAD liposomes. Rabbits were immunized with 20 μg of Pfs230C1 with CoPoP/PHAD liposomes (80 μg CoPoP and 32 μg PHAD) on day 0 and day 28, final bleeding was collected on day 56. **a** Anti-Pfs230C1 IgG ELISA data and (**b**) SMFA functional assay. ELISA experiment were performed with *n* = 4 of independent rabbits and line represents geometric mean. SMFA experiments were performed with *n* = 20 mosquitoes and lines show arithmetic mean. For (**a**), log-transformed titer was compared based on a paired Student’s *t* test. ***p < 0.0005; ****p < 0.0001.

Based on the encouraging results observed in mice, we next assessed Pfs230C1 immunization with CoPoP/PHAD liposomes in rabbits. The motivation for this was to show the efficacy of the adjuvant system in a second species, and rabbits are a larger animal with a different immune system compared to mice. For example, rabbit TLR-4, the target of PHAD, has more similarity to human TLR-4, suggesting that rabbits might be better used to model human immune responses^32^. Rabbits were immunized with 20 μg of Pfs230C1 with CoPoP/PHAD liposomes intramuscularly on day 0 and day 28. The post-immune serum showed strong anti-Pfs230C1 IgG titer on day 28 and followed by boosting effect on day 56 (Fig. 7a). Purified IgG from the post-immune sera from individual rabbits produced a full transmission-blocking response (Fig. 7b), showing that CoPoP/PHAD induced strong immunogenicity in both mice and rabbits (Supplementary Table 3).

## Discussion

In this work, we first confirmed that Pfs230C1 forms particles after simple admixing with CoPoP liposomes. Particle formation is based on the short his-tag sequence of the protein binding with the chelated cobalt of the porphyrin moieties in the bilayer. Antibodies with greater transmission-reducing function were induced with CoPoP/PHAD relative to Alum in mice. Studies have shown that Alum generally induces Th2-biased immunity responses^33^, while CoPoP/PHAD and ISA720 induced a higher ratio of IgG2-to-IgG1 antibodies, reflective of a Th1-biased response. A previous study has shown that the IgG2-to-IgG1 ratio is important for Pfs230C1 antibody function in the SMFA^11^ and this study demonstrated that IgG2 subclass was substantially greater in ISA720 and CoPoP/PHAD adjuvants. It is also possible that other factors, such as the specific antibody epitope response varied based on antigen presentation by the different adjuvants. When comparing CoPoP/PHAD and Alum adjuvants, that produced similar antibody levels, the IgG2-to-IgG1 ratio could explain the improved results of CoPoP/PHAD liposomes. However, for comparing the CoPoP/PHAD and ISA720 adjuvants, which produced similarly high IgG2-to-IgG1 ratios, the higher levels of antibodies induced by ISA720 could explain differences in SMFA activity. As mentioned above, ISA720 is a potent vaccine adjuvant that has been used in clinical studies. However, in one study, injection reactions were observed in 24% of the patients^34^. Although reactogenicity was not studied in this work, a prior study in mice found that CoPoP/PHAD with Pfs25 had minimal local reactogenicity compared to ISA720^18^.

Pfs230C1 is generally an effective immunogen, while Pfs25 has poor immunogenicity due to its putative hapten-like behavior^35^. In our previous study, we found that nanogram doses of Pfs25 induced strong immunogenicity with the CoPoP/PHAD platform^18^, but not with other adjuvants including ISA720 or Alum. The present study sheds additional light on these observations and shows that Pfs230C1, unlike Pfs25, could induce antibodies and be taken up by APC in vitro and in vivo with or without particle formation. The mechanism for the enhanced uptake of soluble Pfs230C1 in immune cells relative to Pfs25 is unclear. CoPoP/PHAD with Pfs230C1 generated efficacious transmission-reducing activity with nanogram antigen doses, a durable antibody response, and a high IgG2-to-IgG1 ratio. CoPoP/PHAD offers other features such as uniform antigen conformation and presentation on the surface of the liposomes and straightforward capability for multiplexing.

In summary, Pfs230C1 could be admixed with CoPoP liposomes to induce antigen particleization. Immunization with CoPoP/PHAD generated functional antibodies in mice using nanogram antigens doses. Enhanced levels of functional antibodies were induced with CoPoP/PHAD relative to Alum, along with higher IgG2-to-IgG1 antibody ratios. Altogether, these results confirm that the CoPoP technology can effectively serve as a particle-inducing vaccine adjuvant for TBV development, resulting in strong and durable induction of functional antibodies.

## Methods

### Materials

His-tagged Pfs230C1 was produced in a baculovirus system as previously reported^10^. CoPoP was produced as previously described^26^. The following adjuvants were obtained: Montanide ISA720 (SEPPIC) and Alhydrogel 2% aluminum gel (Accurate Chemical and Scientific Corporation; Cat #A1090BS). The following lipids were used: 1,2-dipalmitoyl-sn-glycero-3-phosphocholine (DPPC, Corden Cat #LP-R4-057), cholesterol (PhytoChol, Wilshire Technologies), synthetic monophosphoryl lipid A Phosphorylated HexaAcyl Disaccharide (PHAD, Avanti Cat #699800) and PHAD-504 (Avanti Cat #699810).

### Antibodies for flow cytometry

For antigen uptake into immune cells in draining lymph nodes, the following antibodies were obtained from Biolegend: I-A/I-E Pacific Blue (Clone: M5/114.15.2; Cat #107619; Lot: B252426), CD11c APC (Clone: N418; Cat #117310; Lot: B253461), F4/80 PE (Clone: BM8; Cat #123109; Lot: B251636).

### Liposome preparation

Liposomes were prepared by ethanol injection and nitrogen-pressurized lipid extrusion in phosphate buffered saline (PBS) carried out at 60 °C followed by dialysis to remove ethanol. Final liposome concentration was adjusted to 320 μg/mL PHAD and samples were passed through a 0.2 µm sterile filter and stored at 4 ° C. Liposome sizes and polydispersity index were determined by dynamic light scattering with a NanoBrook 90 plus PALS instrument after 200-fold dilution in PBS.

The standard CoPoP/PHAD liposome formulation had a [DPPC:Chol:PHAD:CoPoP] mass ratio of [4:2:1:1]; CoPoP liposomes used a [DPPC: Chol: CoPoP] mass ratio of [4:2:1], and PoP/PHAD liposomes used a [DPPC: Chol: PHAD: PoP] mass ratio of [4:2:1:1]. For liposome preparation, ethanol and PBS were preheated at 60 °C in the water bath. Dry lipids were weighed and dissolved in 1 mL ethanol and sonicated briefly (~5 s) in a water bath sonicator to break up large particles. After 10 min of incubation at 60 °C, 4 mL of PBS was added to the liposome solution and incubated for another 10 min. A nitrogen-pressurized liposome extruder (Northern Lipids) was preheated to 60 °C, and a membrane stack of 200, 100 and 80 nm polycarbonate filters was placed into the extruder and rinsed with distilled water. The liposome solution (1 mg/mL CoPoP or equivalent) was extruded 15 times at 60 °C with a pressure near 200 PSI through the membrane stack. To remove ethanol from liposomes, the samples were then dialyzed in 500 mL of PBS for 4–6 h at 4 °C. The buffer was changed and further dialyzed overnight at 4 °C. The liposomes were diluted to 320 μg/mL CoPoP (or equivalent) in PBS and then passed through a 0.2 μm sterile filter in a biological safety cabinet. Liposome size and polydispersity index were determined by dynamic light scattering in a NanoBrook 90 plus PALS instrument after samples were diluted 200-fold in PBS.

### Antigen-binding characterization

Liposome binding with Pfs230C1 was generally carried out by incubating protein and liposomes with a 1:4 mass ratio of protein:CoPoP. Following incubation, the sample was subjected to microcentrifugal filtration (PALL Cat #29300) and protein in the filtrate was assessed by micro BCA (Thermo Cat #23235). For gel electrophoresis, loading dye (50% glycerol, Tris-HCl (0.25 M, pH 6.8) and 0.25% bromophenol blue) was added to liposome samples and 1.5 μg of Pfs230C1 with indicated liposomes were loaded into a native Tris-Glycine PAGE gel (Lonza Cat #8522) and subjected to electrophoresis and staining. CoPoP/PHAD, CoPoP and PoP/PHAD liposomes were admixed with Pfs230C1. For CoPoP/PHAD and CoPoP liposomes, Pfs230C1 and liposomes were mixed with 1:1, 1:2, 1:3 or 1:4 mass ratio of protein: CoPoP. Protein without liposomes was used as a control.

To determine binding saturation, 150 μL of Pfs230C1 (80 μg/mL) was mixed with 150 μL of liposome containing 320 μg/mL of CoPoP or PoP in PBS for 3 h at room temperature. Samples were placed in a 100 kDa cut-off microcentrifugal filtration tube pre-rinsed with PBS and centrifuged at 1200 g for 60 min. The flow through was collected and analyzed with micro BCA assay to detect the amount of unbound proteins. Pfs230C1 binding to CoPoP liposomes or PoP liposomes was determined by measuring the BCA absorbance at 562 following manufacturer’s protocol, using the following equation:

$$\% {\mathrm{Pfs}}230{\mathrm{C}}1\,{\mathrm{binding}} = (1 - OD562_{{\mathrm{filteredCoPoP}}/{\mathrm{PHAD}} + {\mathrm{Pfs}}230{\mathrm{C}}1}/{\mathrm{OD}}562_{{\mathrm{filtered}}\,{\mathrm{Pfs}}230{\mathrm{C}}1}) \times 100\%$$

For native PAGE, loading dye was prepared containing 50% glycerol, Tris-HCl (0.25 M, pH 6.8) and 0.25% bromophenol blue. Loading dye was mixed with the incubated samples and loaded into the gel. A Tris/Glycine buffer was used to run the gels (Biorad, Cat # 1610734). The samples were subjected to electrophoresis at 200 mV for 35 min. The gel was stained with Coomassie staining buffer (0.1% Coomassie Brilliant Blue G-250, 50% methanol and 10% acetic acid) for 30 min and destained with destaining buffer (40% methanol, 10% of acetic acid in deionized water) with overnight shaking at room temperature.

### Labeling Pfs230C1 with fluorescent dyes

Pfs230C1 was labeled with oyster-488 tetrafluorphenylester (oyster-488, OY-488-T). Labeling was carried out with oyster-488 to Pfs230C1 molar ratio of 10:1. 150 µg of Pfs230C1 was dialysis into 100 mM sodium bicarbonate buffer (pH 9) for 4–6 h at 4 °C twice, and then labeled with oyster-488. Free dye was removed by dialysis against PBS.

Pfs230C1 was also labeled with DY-405-NHS-Ester (DY-405). Labeling was carried out with DY-405 to Pfs230C1 molar ratio of 5:1. 100 µg of Pfs230C1 was dialyzed in 100 mM sodium bicarbonate buffer (pH 9) for 4–6 h at 4 °C twice, and then labeled with DY-405 at room temperature for 1 h. Free dyes was removed by dialysis against PBS three times at 4 °C.

Pfs25 was labeled with DY-490-NHS-Ester (DY-490). Labeling was carried out with a DY-490 to Pfs25 molar ratio of 5:1. 100 µg of antigen was dialyzed into 100 mM sodium bicarbonate buffer (pH 9) at 4 °C and then labeled with DY-490 for 1 h at room temperature, followed by dialysis against PBS three times at 4 °C to remove free dye.

### Cryo-electron microscopy

Pfs230C1 (80 µg/mL) was mixed with an equal volume of CoPoP/PHAD liposomes (320 µg/mL PHAD) in PBS. Reaction mixtures were incubated at room temperature for 3 h and then stored on ice until applied to the electron microscopy grids. Holey carbon grids (c-flat CF-2/2-2C-T) with a freshly applied continuous thin carbon layer were washed with chloroform. Then, grids were glow discharged at 5 mA for 15 s immediately before the application of the sample. A volume of 3.6 µL of sample was deposited in the grid and vitrification was performed in a Vitrobot (ThermoFisher) by blotting the grids once for 3 s and blot force +2 before they were plunged into liquid ethane. Temperature and relative humidity during the vitrification process were maintained at 25 °C and 100%, respectively. The grid was loaded into the Tecnai F20 electron microscope operated at 200 kV using a Gatan 626 single tilt cryo-holder. Images were collected in a Gatan Ultrascan 4000 4k x 4k CCD Camera System Model 895 at a nominal magnification 60,000×, which produced images with a calibrated pixel size of 1.8 Å/pixel. Images were collected with a total dose of ~50 e^−^/Å^2^ using a defocus ranging from −2.7 to −3.5 μm. Images were cropped and prepared for figures using Adobe Photoshop.

### Murine immunization and serum analysis

Five to six week old female CD-1 mice (Envigo) received intramuscular injections on days 0 and 21 containing the indicated antigen doses combined with the indicated adjuvants. Serum was collected on day 42 unless otherwise indicated and sent to the Laboratory of Malaria and Vector Research at the National Institute of Allergy and Infectious Diseases (NIAID) for analysis, which was carried out as previously described for anti-Pfs230C1 ELISA^36^, SMFA^37^, antibody avidity measurement^38^, and antibody subclass analysis^10^. The SMFA was performed at 750 µg/mL of purified total IgGs in the presence of human complement.

### Rabbit immunization

Ten to twelve week old New Zealand white female rabbits received intramuscular injections on days 0 and 28 of 20 µg of Pfs230C1 with CoPoP/PHAD liposomes. Rabbit studies used liposomes made with PHAD variant PHAD-504. Liposomes were prepared with [DPPC: Chol: PHAD-504: CoPoP = 4: 2: 0.4: 1] mass ratio. Serum was collected on day 0, 28 and day 56 and sent to NIAID for ELISA and SMFA analysis. The SMFA was performed at 3750 µg/mL of purified total IgGs in the presence of human complement.

### Flow cytometry

Flow cytometry studies were carried out using a BD LSRFortessa^TM^ X-20 cytometer. Flowjo (version 10) software was used for data analysis. FACS buffer was prepared with 0.5% bovine serum albumin (BSA) and 0.05% sodium azide in PBS.

### Cellular uptake studies

RAW264.7 murine macrophage cells were obtained from ATCC and cultured in in Dulbecco’s modified Eagle’s medium (DMEM) with 10% fetal bovine serum (FBS) and 1% penicillin/streptomycin at 37 °C. For in vitro uptake studies, RAW264.7 cells were cultured in a 24-well plate overnight to reach 70–80% confluence. Cells were then treated with liposomes with 1 µg/mL of Pfs25-DY490 or Pfs230C1-DY405. After incubation for 4 h, cells were harvested and washed with ice-cold PBS 3 times. To dislodge cells, 0.5 mL of FACS buffer was added to each well, and a cell scraper to gently collect the cells.

For draining lymph node studies of Pfs230C1 uptake in immune cells, mice were intramuscularly immunized in the femoral muscle with 1 µg of Pfs230C1-oyster488 conjugate. Two days after injection, mice were euthanized and inguinal lymph nodes were collected. Cells were extracted and fixed with 4% paraformaldehyde at room temperature for 15 min, washed three times with 3 mL PBS by centrifugation at 500 rcf for 5 min. 5 × 10^5^ cells per tube were stained for 30 min at room temperature with murine antibodies against I-A/I-E, B220, CD11c or F4/80 (BioLegend) in FACS buffer.

### Indirect immunofluorescence assay (IFA)

Stage V gametocyte cultures were smeared into thin films and after drying, the slides were fixed with 1:1 methanol:acetone at 4 °C for 30 min. After blocking, the slides were incubated overnight at 4 °C with primary antibodies (1:500 dilution). Next, the slides were incubated with an Alexa Fluor 568 secondary antibody (1:500 dilution) for 1 h at room temperature. Finally, prolong glass with NucBlue was added to stain nucleic acids and mount coverslips. All images were captured on a Lecia SP8 confocal microscope equipped with a white light laser running LAS X software version 3.5.5.19976. Images were captured at a magnification of 63x with a resolution of 1024 × 1024 pixels, deconvolved using Huygens essential software version 19.04.0p2 64b and viewed on Imaris version 9.5.0.

### Statistical analysis

Statistical tests were performed as described in figure captions using Graphpad 6.0, with the exception of comparisons of SMFA groups, which was analyzed by a zero-inflated negative binomial model as previously described^39^. IgG2-to IgG1 ratio were calculated as followed: OD value(IgG2a + IgG2b + IgG2c) /OD valueIgG1. Percentage of TRA were calculated as followed: 100 ×[(mean number of oocysts in the test)/(mean number of oocysts in the control)], and LMR is calculated based on Log10[100/100-percentage of TRA]. Linear regression analysis for anti-Pfs230C1 IgG level in feeder (square root) to LMR were measured by signal linear regression analysis by Graphad 6.0.

### Ethics statement

All mice experiments were carried out using protocols approval of University at Buffalo Institutional Animal Care and Use Committee (IACUC). All rabbit experiments were carried out using protocols approved by the Pocono Rabbit Farm IACUC.

### Reporting summary

Further information on experimental design is available in the Nature Research Reporting Summary linked to this article.

## Supplementary information

Supporting Information

Reporting Summary

## Data Availability

All raw data are available upon request.

## Code availability

The RStudio code used to plot 3D figures in Fig. 5a is available in the supplementary data.
