# Supplementary material for: Antibody response of a particle-inducing, liposome vaccine adjuvant admixed with a Pfs230 fragment
Source: NPJ Vaccines. 2020 Mar 18;5:23. doi: 10.1038/s41541-020-0173-x (PMC7080793; doi:10.1038/s41541-020-0173-x)
Supplement: Supplementary file 1 — Supporting Information [file 41541_2020_173_MOESM1_ESM.pdf]

Supporting Information: Antibody Response of a Particle-Inducing, Liposome Vaccine Adjuvant Admixed with a Pfs230 Fragment

Wei-Chiao Huang, Bingbing Deng, Amal Seffouh, Joaquin Ortega, Carole Long, Ragavan V. Suresh, Xuedan He, Kazutoyo Miura, Shwu-Maan Lee, Yimin Wu, Jonathan F. Lovell

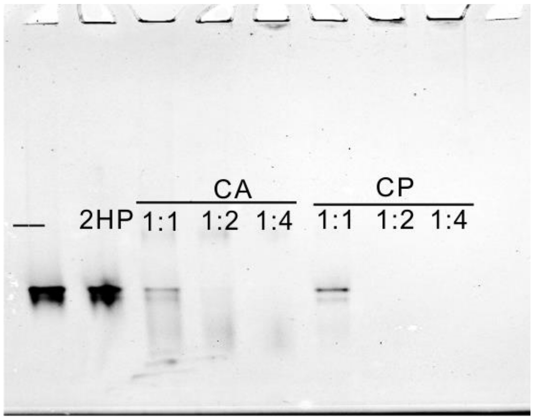

Supplement Figure 1. Native PAGE of Pfs230C1 after 1 hr incubation with indicated liposomes. Under native conditions, the protein is separated based on not only their size, but also their charge. No molecular weight marker is included.

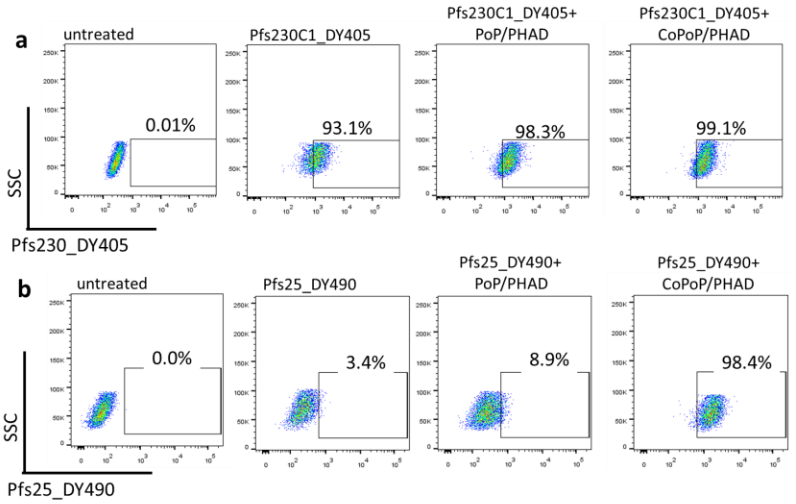

Supplement Figure 2. Gating for RAW264.7 macrophages for fluorophore-labeled antigen uptake. a, Pfs230C1 and **b**, Pfs25 were admixed with CoPoP/PHAD, PoP/PHAD or PBS. The antigen positive cells were gated compared to untreated cells, from n=3 independent experiments.

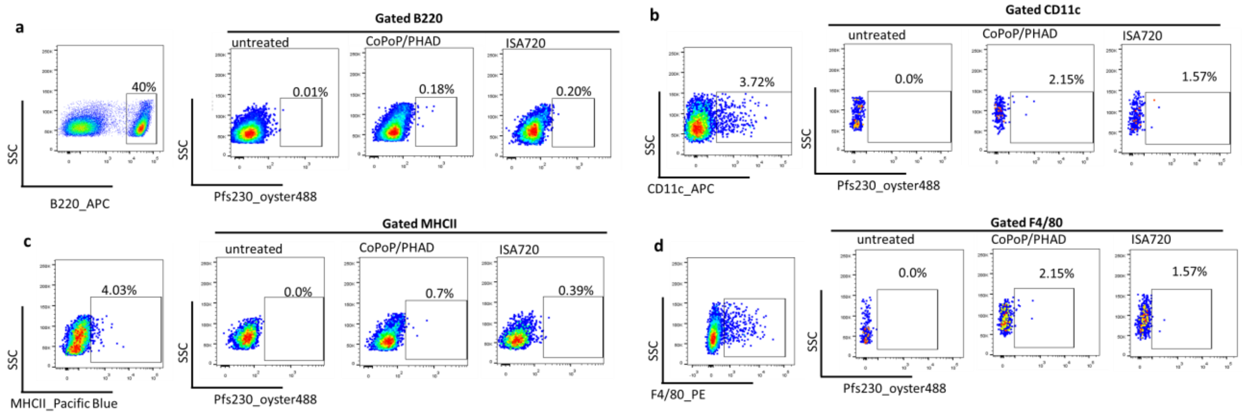

Supplement Figure 3. Gating for APC cells uptake of fluorophore-labeled Pfs230C1 in draining lymph nodes. Cells were first gated by SSC-FSC then **a,** B220, **b,** CD11c, **c,** MHCII and **d,** F4/80 positive cells were gated compared to unstained cells. Pfs230-positive cells were gated with Oyster488-labeled Pfs230C1. Representative plots are shown from biologically independent experiments with n=3 mice.

Supporting Tables of SMFA results

Each SMFA test group (i.e. each table entry) was assessed with n=20 mosquitos per group. Table entries indicate the sample dose used for immunization. Statistical testing is based on a zero-inflated negative binomial random effects model that has been described previously (Miura et al. Vaccine. 2016;34:4145-51).

Supplementary Table 1. Summary of ELISA data

**Sample condition**			**Day**	**Serum**	**Purified IgGs**				**SMFA**					
Study #	Adjuvant	Pfs230 dose [ng]	Day	Geo Mean [EU]	Conc [mg/mL]	Total EU	IgG2/1 Ratio	Avidity IC50 [M]	SMFA#	Test IgG [ug/mL]	% TRA	p-value	LMR	EU in feeder [SQRT]
MA0068-1	CoPoP/ PHAD	1000	42	47,879	4	87,670	4.68	4.43	182-1	750	99.1	0.001	2.04	128.21
	CoPoP/ PHAD	100	42	4,085	4	15,381	3.85	4.27	182-1	750	84.4	0.001	0.81	53.70
	CoPoP/ PHAD	10	42	19,775	4	43,879	5.42	3.86	182-1	750	99.1	0.001	2.04	90.70
	CoPoP alone	1000	42	15,986	4	35,831	0.77	4.01	182-1	750	49.5	0.116	0.30	81.97
	PoP/ PHAD	1000	42	30,399	4	59,717	2.68	3.25	182-1	750	63.3	0.012	0.44	105.82
	Alum	1000	42	26,471	4	66,893	0.57	4.60	182-1	750	79.8	0.001	0.70	111.99
	ISA720	1000	42	156,547	4	211,271	2.92	4.38	182-1	750	100.0	0.001	Inf*	199.03
MA0068-2	CoPoP/ PHAD	125	42	30,294	4	64,308	3.53	4.96	192-1	750	99.8	0.001	2.80	109.81
	CoPoP/ PHAD	10	42	14,335	4	26,446	3.90	4.62	192-1	750	93.2	0.001	1.17	70.42
	CoPoP/ PHAD	0.8	42	2,198	4	8,214	2.59	3.97	192-1	750	28.4	0.439	0.15	39.24
	ISA720	125	42	74,222	4	96,923	4.87	4.70	192-1	750	100.0	0.001	Inf*	134.81
	ISA720	10	42	30,201	4	68,528	6.60	4.33	192-1	750	100.0	0.001	Inf*	113.35

	ISA720	0.8	42	84	4	1,604	9.14	3.87	192-1	750	51.7	0.069	0.32	17.34
	alum	125	42	29,265	4	72,396	0.85	4.31	192-1	750	92.3	0.001	1.11	116.51
	ISA720	200	42	87,356	2	49,049	2.89	4.59	205-1	750	99.7	0.001	2.50	135.62
	ISA720	40	42	28,917	2	27,803	4.85	4.28	205-1	750	100.0	0.001	Inf *	102.11
	Alum	5000	42	43,314	2	34,698	0.87	4.82	205-1	750	60.7	0.019	0.41	114.07
MA-0078-00	Alum	1000	42	13,651	2	19,525	1.12	4.28	205-1	750	67.0	0.022	0.48	85.57
	CoPoP/ PHAD	50	42	17,297	2	17,413	4.33	5.95	205-1	750	91.1	0.001	1.05	80.81
	CoPoP/ PHAD	10.0	42	5,854	2	5,271	4.11	5.68	205-1	750	27.4	0.420	0.14	44.46
	CoPoP/ PHAD	2.0	42	2,481	2	4,589	3.17	6.85	205-1	750	23.5	0.484	0.12	41.48
	ISA720	200	42	80,172	2	66,874	1.93	4.36	205-1	750	98.8	0.001	1.91	158.36
	ISA720	40	42	2,908	2	29,090	3.76	4.23	205-1	750	89.7	0.001	0.99	104.44
	Alum	5000	42	28,854	2	28,368	0.54	4.51	205-1	750	54.2	0.061	0.34	103.14
PC-067-00	Alum	1000	42	15,722	2	24,993	0.40	4.24	205-1	750	62.8	0.019	0.43	96.81
	CoPoP/ PHAD	50	42	15,584	2	16,153	3.96	5.19	205-1	750	63.8	0.018	0.44	77.83
	CoPoP/ PHAD	10	42	7,675	2	11,870	2.49	5.43	205-1	750	73.1	0.001	0.57	66.72
	CoPoP/ PHAD	2	42	548	2	1,259	5.61	4.81	205-1	750	39.2	0.223	0.22	21.73
	Alum	15000	42	54,377	4	82,917	0.49	4.25	233-2	750	45.0	0.187	0.26	124.69
	Alum	1000	42	22,958	4	40,736	0.97	4.10	233-2	750	71.2	0.004	0.54	87.40
	ISA720	200	42	100,369	4	128,635	4.45	4.16	233-2	750	100.0	0.001	Inf*	155.30
	ISA720	50	42	21,627	4	55,400	5.55	4.21	233-2	750	92.5	0.001	1.13	101.92
	CoPoP/ PHAD	200	42	21,353	4	46,708	6.20	4.95	233-2	750	90.2	0.001	1.01	93.58
MA-0086-00#	CoPoP/ PHAD	50	42	17,025	4	40,799	5.45	5.20	233-2	750	97.4	0.001	1.59	87.46
	Alum	5000	42	46,210	4	78,563	0.51	4.20	233-2	750	-15.7	0.738	-0.06	121.37
	Alum	15000	250	4,634	8	9,358	1.05	4.65	248-2	750	1.4	0.977	0.01	29.62
	Alum	1000	250	8,913	8	11,507	1.14	4.53	248-2	750	46.8	0.142	0.27	32.85
	CoPoP/ PHAD	200	250	12,963	8	22,885	14.63	5.89	248-2	750	-7.8	0.881	-0.03	46.32
	CoPoP/ PHAD	50	250	15,201	8	34,405	6.83	4.93	248-2	750	66.0	0.008	0.47	56.79

Table entries show serum geometric mean (Geo Mean) in ELISA units (EU), IgG concentration, total ELISA units, IgG2-to-IgG1 ratio, antibody avidity IC50 (based on urea denaturation), standard membrane feeding assay (SMFA) test number, the transmission reducing activity (TRA) and the log-mean-ratio (LMR) the log10 of the ratio of the mean number of oocysts in the SMFA control to the mean number of oocysts in the test sample.

**Inf = infinity*

#Some immunizations in this study were done in duplicate at two sites

Supplementary Table 2. Comparison between Pfs25 and Pfs230C1 with the combination of CoPoP/PHAD or ISA720

**Adjuvant**	**CoPoP/PHAD**	**ISA720**	**CoPoP/PHAD**	**ISA720**
Antigen	Pfs25 (100 ng)	Pfs25 (100 ng)	Pfs230C1 (125 ng)	Pfs230C1 (125 ng)
	MA-0056		MA-0068	
Purified IgG 4 mg/ml	122036	12139	64307.5	96922.7
	SMFA-170_2		SMFA-192	
% inhibition	92.3	-87.2	99.8	100

Supplementary Table 3. SMFA #251 (rabbit)

Sample	IgG conc [mg/ml]	Oocyte Av.	% inhibition estimate	p-value
Rabbit #1	3.75	0.1	99.1	0.001
Rabbit #2	3.75	0.0	100	0.001
Rabbit #3	3.75	0.1	99.1	0.001
Rabbit #4	3.75	0.0	100	0.001
Control: Pre-immune sera	3.75	5.3	--	--

<RStudio code>

The RStudio code were used to plot the data in **figure 5a**, showing x axis as IgG2-IgG1 ratio, y axis as percentage of transmission-reducing activity and z axis as Pgs230C1 IgG level, coding details could be found below:

```
install.packages("scatterplot3d", dependencies = TRUE)

library(scatterplot3d)

scatterplot3d(Data2[,1:3])


scatterplot3d(Data2[,1:3], angle = 40)

scatterplot3d(Data2[,1:3],

        main="3D Scatter Plot",

        xlab = "IgG subclass",

        ylab = "% TRA",

        zlab = "EU")

shapes = c(15, 16, 17)

shapes <- shapes[as.numeric(Data2$G)]

scatterplot3d(Data2[,1:3], pch = shapes)

colors <- c("#999999", "#0000FF", "#56B4E9")

colors <- colors[as.numeric(Data2$G)]

scatterplot3d(Data2[,1:3], pch = 16, color=colors)


scatterplot3d(Data2[,1:3], pch = 16, color = colors,

        grid=TRUE, box=FALSE)

addgrids3d(x, y=NULL, z=NULL, grid = TRUE,

      col.grid = "grey", lty.grid=par("lty"))

scatterplot3d(Data2[,1:3], pch = 16, type="h",

        color=colors)

s3d <- scatterplot3d(Data2[,1:3], pch = 16, color=colors)

legend(s3d$xyz.convert(7.5, 3, 4.5), legend = levels(Data2$G),

    col =  c("#999999", "#E69F00", "#56B4E9"), pch = 16)
```

```
s3d <- scatterplot3d(Data2[,1:3], pch = 16, color=colors)
legend("right", legend = levels(Data2$G),
       col =  c("#999999", "#E69F00", "#56B4E9"), pch = 18)

s3d <- scatterplot3d(Data2[,1:3], pch = shapes)
legend("bottom", legend = levels(Data2$G),
       pch = c(16, 17, 18),
       inset = -0.25, xpd = TRUE, horiz = TRUE)
s3d <- scatterplot3d(Data2[,1:3], pch = 16, color=colors)
legend("bottom", legend = levels(Data2$G),
       col =  c("#blue", "#E69F00", "#56B4E9"), pch = 15,
       inset = -0.25, xpd = TRUE, horiz = TRUE)
s3d <- scatterplot3d(Data2[,1:3], pch = shapes, color=colors, cex.symbols=2.7,angle = 65, type="h")
legend("bottom", legend = levels(Data2$G),
       col =  c("#999999", "#E69F00", "#56B4E9"),
       pch = c(15, 16, 17),
       inset = -0.25, xpd = TRUE, horiz = TRUE)
addgrids3d(Data2[, 1:3], grid = c("xy", "xz", "yz"))
library(addgrids3d)
source('~/hubiC/Documents/R/function/addgrids3d.r')
addgrids3d(x, y=NULL, z=NULL, grid = TRUE,
           col.grid = "grey", lty.grid=par("lty"))
```
